# Round the Hospitals

**Published:** 1918-05-18

**Authors:** 


### ROUND THE HOSPITALS.

Great interest is being taken in the democratic election of members on the Council of the College of Nursing. We hope every nurse on the Register will vote, and will take care that her voting paper is in order and is posted before Monday, May 20, in an envelope addressed to the Secretary of the College of Nursing (Limited by Guarantee), 6 Vere Street, London, W. 1. Each nurse who can possibly attend the third annual general meeting on Thursday, June 6, at 3 p.m., at the rooms of the Royal Society of Medicine, 1 Wimpole Street, W., should make a point of doing so. Every nurse has herself a special opportunity to support the interests of the College by taking part in the effort to recruit candidates for registration continuously, up to and including June 6, the date of the annual meeting. It is possible that there may be a very interesting competition amongst nurse-training schools, associations of nurses, co-operations, and private nursing staffs to rally the nurses of each to submit their names for the College Register before June 6, because all trained nurses on the Register must recognise the importance of bringing the actual number registered up to 10,000 or more. As we said last week, this can be readily accomplished, if every registered nurse will make it her business, to secure at least one friend to send in her name. The institutions and nurses' associations have here an opportunity to show their interest in the first democratic election for the College, and their resolve to leave no stone unturned to get on with the Register and the endowment of the College, that trained nursing may speedily be organised on a basis, wide enough to secure the best interests of the nursing profession, and the speedy betterment of the position, emoluments, and status of trained nurses. Our readers will find an interesting letter on this point from "Ierne," which should raise a most helpful discussion and prove fruitful in results (see page 143).

---

This week the nursing profession have an opportunity to contrast the democratic basis of the College election with the discredited non-democratic nomination plan of the Royal British Nurses' Association (see page 144). We have had some amusing experiences since the issue of *The Nurses' Journal* for May, with which is circulated a printed document vaguely alluded to, as "This Voting Paper" and "the Ballot Paper," on which thirty names are printed, and no more. No column is ruled off for the record of the vote by means of the usual cross, and no adequate provision is made for the nomination of other persons, whose names can be entered, should the members desire to substitute other names than the thirty in question. Of these thirty ten are practitioners, ten are described as matrons and superintendents of nurses, and ten as sisters and nurses, for election. On the back of this paper are printed the address of the secretary of the R.B.N.A., and in large type the words "Ballot Paper." This document appears to have excited mixed feelings on the part of those immediately interested in its contents. At first some, indeed, declined to believe that amongst the matrons for election were entered the names of several devoted allies of Mrs. Bedford Fenwick and of that lady herself. The document indicates, as a contemporary bluntly assumes, that the members of the Royal British Nurses' Association have no opinions of their own so far as this election is concerned, and that the arranged programme, including the list of names circulated, will be swallowed whole. Our amusement is due to the astonishment exhibited by old adherents of the R.B.N.A. who have been associated with its history since the commencement, who remember the fate of the Lady who went for a Ride on the Tiger. The present are strenuous times, and trained nurses have been grateful to *The Nurses' Journal* for its illuminating gift of a humorous surprise packet with its May issue. The question is being asked, how many independent members of the R.B.N.A. will accept the gracious permission given, to erase from the list any of the names they please, and to substitute an equal number of other names? Dr. A. P. Beddard and Mr. James Berry should escape erasure, as no room is left for the name of a substitute. The merriment created by this production is all to the good, and makes the document one that should be retained and preserved.

---

We are asked to state that the arrangements for the Conference to be held on June 6 and 7, under the auspices of the College of Nursing in connection with its third annual general meeting, are making good progress. The Conference Committee will be glad of offers of hospitality from London residents of a bed for the night of June 6, and of breakfast the following morning. Members who wish to avail themselves of this hospitality are asked to write as soon as possible to Miss Girdlestone, who has kindly undertaken this part of the Conference arrangements, at the offices of the College of Nursing, 6 Vere Street, London, W. 1. On similar occasions of importance to Pension Fund nurses, the hospital authorities throughout, the Metropolis, very generously gave invaluable help in finding beds and hospitality for the members of that Fund. We are hopeful that the same course may be adopted by London hospital authorities, generally, in connection with the third annual general meeting and Conference of the College of Nursing, in which they are for the most part directly and warmly interested.

---

The Overseas Nursing Services have already rendered much valuable and timely help to British nurses, and British gratitude found its best expression in the recent reception accorded by the Queen to the principal matrons of the Canadian, Australian, and New Zealand Army Nursing Services—Miss Margaret Macdonald, R.R.C., Miss Evelyn Conyers, R.R.C., and Miss Mabel Thurstan, R.R.C. The Queen received the Matrons-in-Chief in her private apartments at Windsor Castle. Princess Mary, who is already linked by much personal

**ROUND THE HOSPITALS—(*continued*).**

work with the Red Cross, and Lady Minto, whose labours in India for the extension of the Nursing Service bore fruit in the Association which bears her name, were present on this historic occasion. The *Daily Telegraph*, in commenting on the Queen's understanding of the love of things domestic which characterises the women of the Daughter-lands, has some appreciative words on the fact that "all the nursing of the war has been carried out with a quiet reticence that has concealed the magnitude of the task performed with such splendid efficiency." And it is true that few even among nurses realise the extent of the succour the Overseas nurses have brought to their over-burdened sisters.

---

American women have a reputation for rapid and efficient organisation. It is, therefore, not surprising to learn that the complex business of developing the French and English branches of the American Red Cross Nursing Service is proceeding at all speed, and promises to evolve a powerful and harmonious body of nurses for service with the troops. Miss Carrie M. Hall has been appointed Chief Nurse in this country, and has already taken up work at 40 Grosvenor Gardens, the headquarters of the American Red Cross Nursing Service. As a member of the National Committee she has had unique opportunities for visaging nursing problems from a broad standpoint, and she has had rich experience in war work as Chief Nurse of the Harvard Unit, No. 11 General Hospital, B.E.F., which has been running about a year. We have had the privilege of thoroughly inspecting the Peter Bent Brigham Hospital, Boston, and seeing Miss Carrie M. Hall at work there when she was in charge as Chief Matron. Everything reflected great credit upon herself and her staff, and the teaching of the nurses and its effect upon their training, which we were allowed to test, was remarkably excellent. We also had the pleasure of meeting Miss Hall again when she was in charge of No. 11 General Hospital, B.E.F., when the same efficiency and administrative strength were evident throughout. We are therefore in a position from actual knowledge sincerely to congratulate not only Miss Carrie M. Hall as the Chief Nurse in this country in charge of the American Red Cross Nursing Service, but all who are responsible for her appointment, for, if service and results are to be the guide, no better appointment could have been made. We wish Miss Hall every possible success and shall always welcome every opportunity to co-operate with and help her and all connected with the American Red Cross Nursing Service in every possible way. Nurses are rapidly coming over, and the *personnel* is likely to be continuously multiplied during the next few months. There are already three hospitals in England staffed entirely by the American Red Cross nursing staff—viz., St. Katharine's Lodge, Regent's Park (officers); Baroda House, Kensington Palace Gardens (officers); and the American Red Cross Military Hospital at Liverpool. Two others, one at Paignton and another in London for officers, are at present staffed partly by British and partly by Americans.

---

Miss Julia Stimson has been recently appointed Chief Nurse of the American Red Cross Nursing Service in France. Like Miss Hall, she is a member of the National Committee; she has been over about a year as Chief Nurse of the St. Louis Unit, No. 9 General Hospital, B.E.F. The nurses under her command are responsible for staffing a large and continually increasing number of base hospitals and clearing stations, many of which have been equipped by the American Red Cross. The dark-blue uniforms of the American nurses are likely to become increasingly familiar in the London streets as the summer goes on. It is a pity that the charming white indoor uniform worn by the Red Cross nurses in their own country has to be replaced by grey when on active service. We understand that the authorities are now engaged over the task of equipping a suitable hospital to which American nurses can be transferred when ill, as well as a convalescent home and rest-house for them.

---

A general and very liberal rise of salaries has taken place at the Royal Infirmary, Liverpool, at which institution the committee have for some time had the question of the salaries of the nursing staff under consideration. Probationers, who formerly began at £10 and rose to £16, £18, £25, and £30 in subsequent years, now start at £12, and receive £18, £22, £30, and £35, the last-named amount for nurses on the private staff in their fifth year being supplemented by 10 per cent. on earnings. War sisters, who in past years received £30 and £35, had their salaries raised early last year to £35, £40, and £45; they have now received a further augmentation of pay to £40, £45, and £50. The out-patients', theatre, and maternity sisters are now to receive £50 from the first. The night sister is to receive £50 to £55, instead of £45 to £50; the massage sister, £100 instead of £50 to £60; the assistant home sister, £45 to £50; the senior home sister, £50 to £60; the matron's assistant, £60 to £70. Miss Cummins, lady superintendent and matron, is to be cordially congratulated on these alterations, which have the effect of placing both higher and lower nursing posts in the institution on a sound pecuniary basis. They remove the objection sometimes urged against continued service in hospitals, that with all its absorbing interest, it gives little scope for making provision for the future.

---

Many indications are forthcoming that the upward trend of salaries is continuing and is likely to be permanent. The managers who give increases in the form of "war bonuses," implying that the old scale will be resumed with the arrival of peace, are in the minority. The new Tuberculosis Hospital at Canons Ware has had to raise its salaries almost as soon as it opened, for the very

**ROUND THE HOSPITALS**—(*continued*).

sufficient reason that the managers were unable to procure nurses at the advertised rates. Sisters' salaries have been raised from £40 to £52, with allowance of £5 a year for uniform. Assistant nurses rise from £28 to £35, probationers from £15 to £20, with an annual increment of £2 to £24. Again from Leeds comes the announcement that staffing difficulties having arisen, especially as regards women inspectors, the following scale has been agreed upon: Grade I, Health Visitors, including clinic nurses who possess a certificate of three years' training, together with the C.M.B., the Royal Sanitary Institute, and the Health Visitor's certificates, £110, rising by annual increments of £10 to £130. Health Visitors possessing the above three certificates but who are not trained nurses are to receive £104, rising by annual increments of £5 to £120.

Probationers at the Bradford Poor-Law Infirmary, who now receive but £10 in their first year, with a £5 war bonus, are not to have their salaries raised. This is the outcome of a debate on the subject, in which several of the Guardians pleaded for Bradford to come into line and advance the salaries at the institution in accordance with what is being done elsewhere. The training-school at the Bradford Union Hospital is a training centre for midwives and enjoys an excellent reputation, while its nursing quarters, with their separate bedrooms, are above the average. We think it will not be long before the Guardians realise that, irrespective of war conditions, salaries have risen all over the country, and that it will not be for the interest of the town to let this fine municipal hospital lag behind.

The handsome gift of £40 subscribed to start the local centre at Plymouth, to which we alluded in our last issue, was the gift of the staff at the 4th Southern General Hospital at Plymouth, and was collected by the Matron, Miss Tait McKay. It does honour to all concerned, and will, we believe, help to set many another ball rolling in the same direction.

**☞ It is our invariable practice to pay for all items of news which we may publish. The minimum payment is 5s. Paragraphs of about twenty lines in length—that is to say, of 150 words or less—are preferred. Contributions should be addressed to The Editor, The Hospital, 28 and 29 Southampton Street, Strand, London, W.C. 2, and, to be in time for the current week's issue, should reach him by the first post on Mondays.**

